# Population and sex differences in *Drosophila melanogaster* brain gene expression

**DOI:** 10.1186/1471-2164-13-654

**Published:** 2012-11-21

**Authors:** Ana Catalán, Stephan Hutter, John Parsch

**Affiliations:** 1 Department of Biology II, University of Munich (LMU), Grosshaderner Str. 2, Planegg-Martinsried 82152, Germany

**Keywords:** Transcriptomics, Adaptation, Population genetics, Insecticide resistance, Sexual dimorphism

## Abstract

**Background:**

Changes in gene regulation are thought to be crucial for the adaptation of organisms to their environment. Transcriptome analyses can be used to identify candidate genes for ecological adaptation, but can be complicated by variation in gene expression between tissues, sexes, or individuals. Here we use high-throughput RNA sequencing of a single *Drosophila melanogaster* tissue to detect brain-specific differences in gene expression between the sexes and between two populations, one from the ancestral species range in sub-Saharan Africa and one from the recently colonized species range in Europe.

**Results:**

Relatively few genes (<100) displayed sexually dimorphic expression in the brain, but there was an enrichment of sex-biased genes, especially male-biased genes, on the X chromosome. Over 340 genes differed in brain expression between flies from the African and European populations, with the inter-population divergence being highly correlated between males and females. The differentially expressed genes included those involved in stress response, olfaction, and detoxification. Expression differences were associated with transposable element insertions at two genes implicated in insecticide resistance (*Cyp6g1* and *CHKov1*).

**Conclusions:**

Analysis of the brain transcriptome revealed many genes differing in expression between populations that were not detected in previous studies using whole flies. There was little evidence for sex-specific regulatory adaptation in the brain, as most expression differences between populations were observed in both males and females. The enrichment of genes with sexually dimorphic expression on the X chromosome is consistent with dosage compensation mechanisms affecting sex-biased expression in somatic tissues.

## Background

The successful colonization of new habitats requires populations to adapt to novel biotic and abiotic conditions. Understanding the basis of this ecological adaptation is a major goal of evolutionary genetics. Because of its demographic history, the fruit fly *Drosophila melanogaster* offers an opportunity to address this fundamental issue in a well-established model system. Presently, *D. melanogaster* has a worldwide distribution spanning a wide variety of habitats. However, biogeographic and population genetic studies indicate that the species has its origin in sub-Saharan Africa and only began to colonize non-African regions about 15,000 years ago [1-6]. The expansion of the species to new, non-tropical environments is thought to have been accompanied by extensive genetic adaptation [4,7-12], although the identification of ecologically adapted genes and the characterization of their functions have proven difficult. Because changes in gene expression are expected to play an important role in adaptation [13-16], transcriptomic studies offer the possibility to bridge the gap between genotypic and phenotypic evolution and identify candidate genes that may have been the targets of regulatory adaptation. With this aim in mind, several microarray studies have been performed to identify gene expression differences between African and non-African *D*. *melanogaster*[17-19]. A comparable study has been carried out in *D*. *simulans*, which has a similar demographic history to *D*. *melanogaster*[20].

Although previous studies identified genes that differ in expression between African and European *Drosophila* populations [18-20], they suffered from some limitations. For example, these studies used mRNA extracted from whole flies. This approach provides a general picture of gene expression averaged over all tissues, but it is biased towards highly expressed genes and those expressed in many (or large) tissues. The use of whole flies typically does not provide the resolution to detect expression changes that occur only in a single tissue. A second limitation to the previous population studies is that they examined flies of only one sex per experiment [18-20]. Because gene expression is highly sexually dimorphic [21], especially when using whole flies or gonads [22], expression profiles can differ greatly between males and females. Consistent with this, there was very little overlap among the genes differing in expression between populations that were identified separately in males and females from whole-fly microarray studies [19], which suggests that most of the between-population expression divergence is sex-specific.

In order to get a more detailed picture of gene expression divergence between African and non-African *D*. *melanogaster*, we performed high-throughput RNA sequencing (RNA-seq) of mRNA isolated from dissected brains of adult males and females from two populations, one from the ancestral species range in sub-Saharan Africa (Zimbabwe) and one from the derived species range in Europe (the Netherlands). We chose to study gene expression variation in the brain because it plays a critical role in processing sensorial input from the environment. The visual, olfactory, and tactile stimuli coming from biotic sources, such as predators and food resources, as well as environmental conditions, such as temperature and humidity, differ greatly between these populations. Many of these environmental stimuli are detected by the sensorial organs of the fly’s head (eyes, antennae, and proboscis) and are then processed by the brain, which produces a specific output that results in a behavioral and/or physiological response. Previous studies have shown that differences in gene expression in the brain can affect traits such as learning, memory, reproductive diapause, lifespan, and foraging behavior [23-26]. Furthermore, many behaviors that vary between strains or populations, including courtship, mating, aggression, and olfactory response, also exhibit sexual dimorphism [27,28].

The goal of this work is to identify genes that differ in their basal levels of brain gene expression between *D*. *melanogaster* strains originating from Africa and Europe. To this end, we use a ‘common garden’ experimental design in which flies from both populations are reared under identical laboratory conditions. This approach detects expression differences that have a genetic basis, but it cannot detect the effects of environment or gene-by-environment interactions. In total we identify 343 genes that differ in expression between the populations and 91 genes that differ in expression between the sexes. Our study represents the first brain-specific comparison of gene expression between African and non-African *D*. *melanogaster* and uncovers many genes that may play a role in ecological adaptation.

## Results

### The *D*. *melanogaster* brain transcriptome

**Figure 1:**
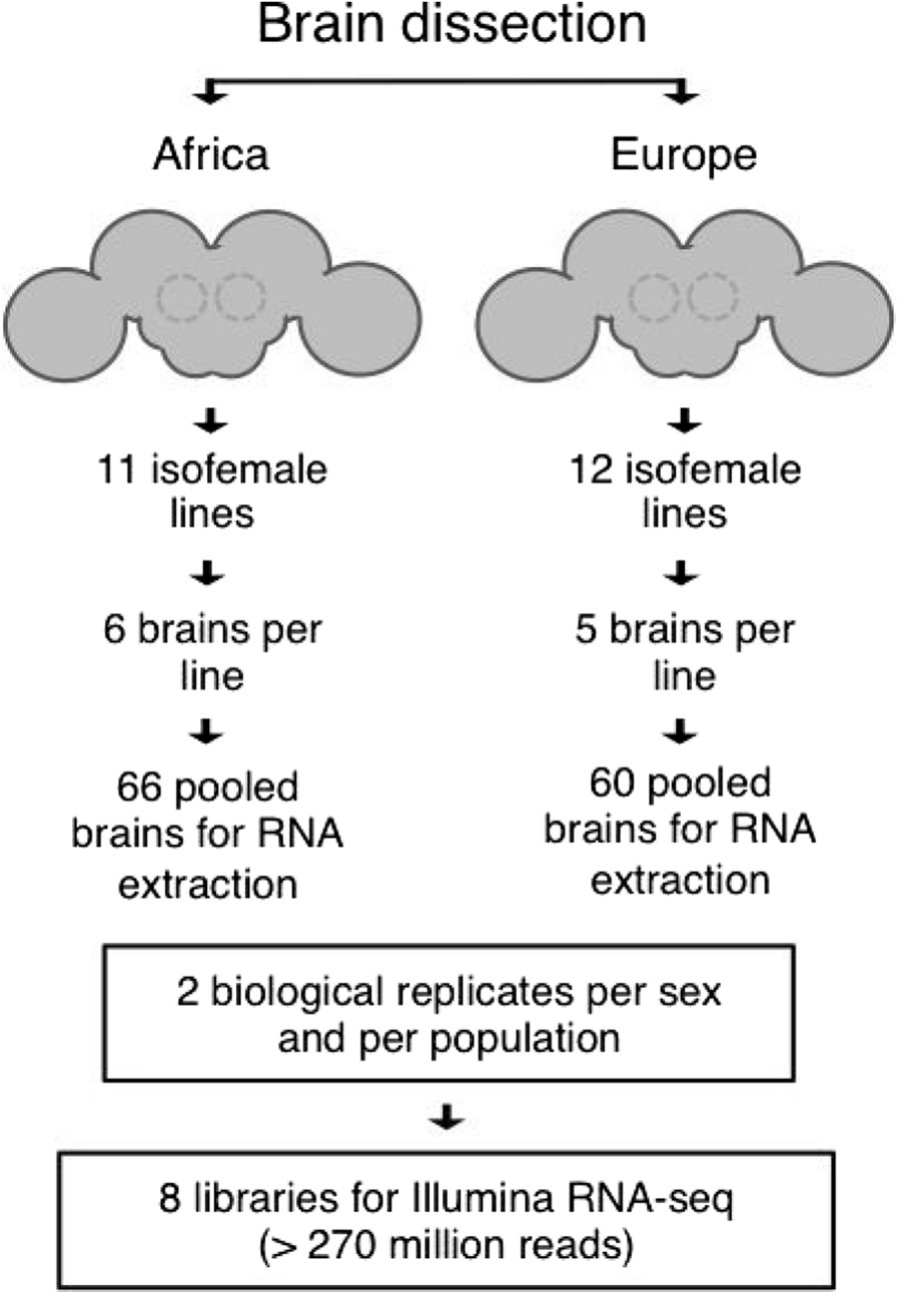
**Overview of RNA sample preparation.** Whole brains were dissected from isofemale lines derived from a European (the Netherlands) and an African (Zimbabwe) population. Brains were pooled within each population for RNA extraction. The dissection and pooling procedures were performed for two biological replicates of each population and sex, resulting in a total of eight samples that were used for cDNA library construction and high-throughput sequencing.

To investigate population differences in gene expression, brains were dissected from 11–12 inbred lines each of an African and a European population (Figure 1). Total RNA was isolated from pooled brains within each population and used to generate cDNA libraries for RNA-seq. In total, we obtained over 270 million short sequence reads from eight cDNA libraries, which included two biological replicates of each sex and population (Table 1). On average, 71% of the reads could be mapped to annotated transcripts. Of the remaining reads, a large proportion (9–20% depending on the library) mapped to ribosomal RNA (rRNA). Because all samples were enriched for poly(A) mRNA before cDNA synthesis, differences in the proportion of rRNA among libraries are likely to reflect differences in mRNA enrichment efficiency. Most of the reads that did not map to transcripts or rRNA could be mapped to intergenic regions (9%) or introns (2%). These may represent unannotated genes or transcript isoforms, but could also result from spurious transcription or intron retention. Around 2% of all reads could not be mapped to the genome (Table 1).

**Table 1:** Number of total and mapped reads (in millions) per sample

		**Mapped reads (%)**			
**Sample**	**Reads**	**Transcripts**	**rRNA**	Other*	**Unmapped (%)**
AfrFemale-R1	24.6	18.7 (75.8)	2.3 (9.2)	3.5 (14.3)	0.20 (0.81)
AfrFemale-R2	44.4	31.6 (71.1)	7.5 (17.0)	5.0 (11.1)	0.37 (0.82)
AfrMale-R1	29.4	20.0 (71.3)	5.0 (16.9)	3.3 (11.1)	0.23 (0.80)
AfrMale-R2	28.6	21.3 (74.5)	3.8 (13.4)	3.0 (10.5)	0.46 (1.60)
EurFemale-R1	27.2	18.3 (67.2)	5.7 (20.7)	2.7 (10.1)	0.54 (1.97)
EurFemale-R2	23.5	16.8 (71.3)	3.8 (16.0)	2.8 (11.8)	0.22 (0.94)
EurMale-R1	48.4	32.3 (66.6)	8.2 (16.9)	4.8 (10.0)	3.16 (6.53)
EurMale-R2	47.3	34.4 (72.7)	6.9 (14.6)	5.2 (11.1)	0.78 (1.64)

*Includes intergenic regions, introns, transposable elements, non-coding RNA (excluding rRNA), and pseudogenes.

**Figure 2:**
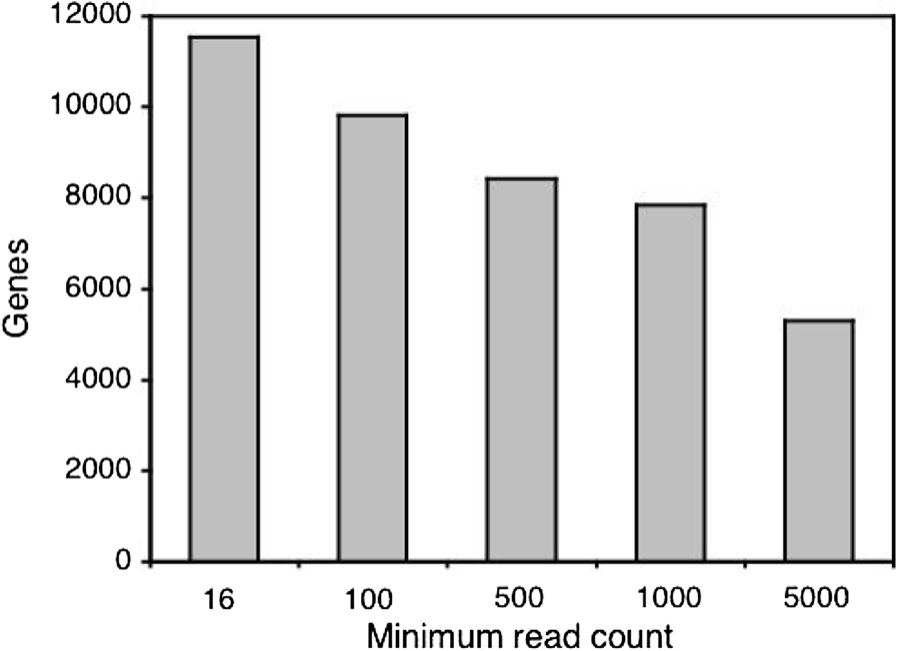
**Number of genes meeting various read-count thresholds.** The Y-axis indicates the number of genes that have the minimum number of mapped reads given on the X-axis. Read counts are summed over all libraries. The set of genes with at least 16 mapped reads was used for analysis of differential expression between sexes and populations.

Of the 13,920 protein-encoding genes annotated in FlyBase release 5.43 [29], 13,575 had at least one mapped read in at least one of the libraries, while 10,873 had at least one mapped read in every library. A total of 11,531 genes had at least 16 reads when summed over all libraries (Figure 2), which was the minimum needed to detect significant differential expression given our experimental design and replication scheme. This set of genes was used for subsequent statistical analyses. Read counts per gene were highly correlated between the biological replicates, with Pearson’s correlation coefficient, *R*, ranging from 0.93 to 0.99.

### Expression differences between the sexes

**Figure 3:**
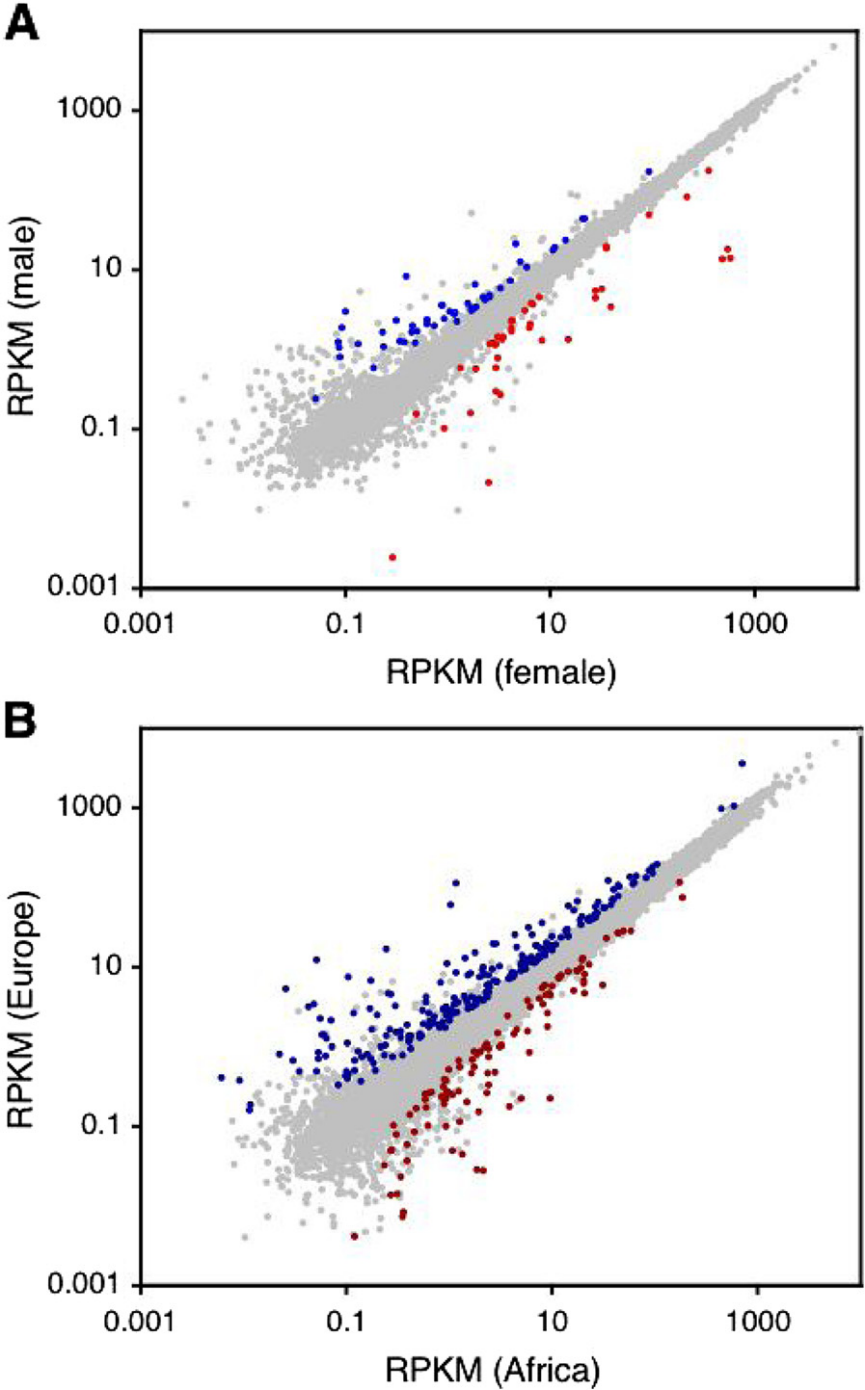
**Comparison of gene expression between sexes and populations.** (**A**) Dot plot of reads per kilobase per million mapped reads (RPKM) from female and male libraries. Genes with significant (FDR<5%) female-biased expression are shown in red. Genes with significant male-biased expression are shown in blue. (**B**) Dot plot of RPKM values from African and European libraries. Genes with significant (FDR<5%) African-biased expression are shown in dark red. Genes with significant European-biased expression are shown in dark blue.

We identified sex-biased genes as those whose expression showed a significant effect of sex in a two-factor analysis that accounted for both sex and population (Figure 3; Additional file 1). Overall, the amount of sexually dimorphic expression was low, with 91 genes showing a significant difference in expression between the sexes at a false discovery rate (FDR) of 5% (Table 2). There was a slight tendency for genes with male-biased expression in the brain to show the same bias in whole flies (Additional file 2). For example, 25 of the 49 genes with male-biased expression in brain also had male-biased expression in whole flies [30,31]. However, of the 24 other genes with male-biased expression in the brain, 16 had female-biased expression and eight had unbiased expression in whole flies. Of the 42 genes with female-biased expression in the brain, 11 had female-biased expression, 10 had male-biased expression, and 22 had unbiased expression in whole flies (Additional file 2).

**Table 2:** Number of sex-biased genes

**Expression**	**Total genes**	**X-linked (%)**	***P***
Sex-biased	91	52 (57)	2x10^-16^
Male-biased	49	39 (80)	2x10^-16^
Female-biased	42	13 (31)	0.017

Enrichment on the X chromosome was tested by Fisher’s exact test.

There was an enrichment of sex-biased genes on the X chromosome, which was significant for both male- and female-biased genes, but much stronger for male-biased genes (Table 2). A previous RNA-seq study using *D*. *melanogaster* heads found a similar enrichment of X-linked sex-based genes and suggested that it was related the mechanism of dosage compensation that occurs on the male X chromosome [32]. To test for a possible influence of dosage compensation on sex-biased expression in the brain, we examined the correlation between the log_2_(male/female) expression ratio of all X-linked genes with at least 100 mapped reads in each sex and the distance to the nearest male-specific lethal (MSL) binding site [33], which represents the assembly point for the dosage compensation complex (DCC). The correlation was significantly negative (Spearman’s *ρ* = −0.11; *P* < 0.001), indicating that genes with relatively high expression in males tend to be close to MSL binding sites. This result held when the minimum read count was increased to 200 or 500 reads per sex. When genes of the different sex-bias classes were compared, male-biased genes were found to be significantly closer to MSL binding sites than female-biased or unbiased genes (Table 3).

**Table 3:** **Distance (*d*) to nearest MSL binding site for X-linked genes**

			**Number of reads with:**		
**Expression**	**Genes**	**Median *d *(bp)**	***d *= 0 bp (%)**	***d *< 3 kb (%)**	***d *< 10 kb (%)**
Male-biased	39	157*	19 (49)	31 (79)**	35 (90)**
Female-biased	13	4,795**	1 (8)	5 (38)	7 (62)
Unbiased	2,089	1,593	771 (37)	1,164 (56)	1,446 (69)

Differences in *d* between male-biased (or female-biased) and unbiased genes were tested by a Wilcoxon test. Differences in the proportion of genes in each category were tested by Fisher’s exact test. *P < 0.01, **P < 0.001.

Because most RNA-seq reads could not be mapped unambiguously to individual transcripts of genes with alternatively spliced isoforms, we had little power to detect sexually dimorphic expression among transcript isoforms. Nonetheless, we did detect significant sexual dimorphism in the expression of *transformer* and *doublesex* isoforms in the brain (Additional file 3). We also detected transcripts of the ribosomal protein genes *RpL17* and *RpS6* that had highly female-biased expression.

### Expression differences between populations

**Figure 4:**
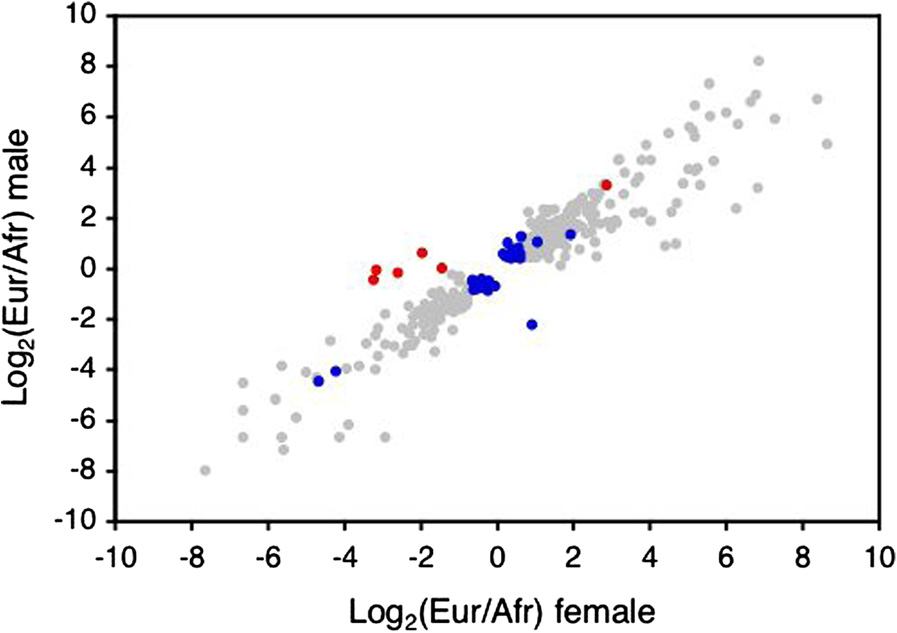
**Between-population expression divergence in females and males.** The ratio of European to African expression (on a log_2_ scale) is plotted for all genes that showed a significant (FDR < 5%) difference in expression between the populations. Gray points represent genes showing a significant effect of population across both sexes. Blue points represent genes that were significant only in males, while red points represent genes that were significant only in females.

We identified genes that differed in expression between the African and European populations as those with a significant effect of population in a two-factor analysis that accounted for both population and sex (Figure 3). This revealed a total of 343 differentially expressed genes at an FDR of 5% (Additional file 1). There were 16 genes that showed a significant effect of both sex and population on their expression (Additional file 1). In all of these cases, the direction of the population bias (European or African) was the same in both sexes. In general, the ratio of European-to-African expression per gene was highly correlated between males and females (Spearman’s *ρ* = 0.63, *P* < 0.0001), indicating that there is little sex-dependent divergence in brain expression between populations. To further investigate this, we analyzed between-population expression divergence separately in females and males using a one-factor (population) analysis within each sex. This revealed 48 genes that were differentially expressed between the populations in one sex, but not the other, and were not detected in the two-factor analysis. The vast majority of these genes (42 out of 48) showed differential expression only in males, which is in contrast to the results previously reported for whole flies [19]. However, even among these genes there was a strong correlation between the European-to-African expression ratios observed in males and females (Spearman’s *ρ* = 0.63, *P* < 0.0001; Figure 4), which again indicates that there is little sex-dependent gene expression divergence in brain between the populations.

There were more genes that showed relative over-expression in the European population (232) than in the African population (111; sign test, *P* < 0.0001). This could result from the mapping of RNA-seq reads to the reference genome (which was generated from a non-African lab strain) being more efficient for the European sample than for the African sample. To test for such a bias in mapping efficiency, we applied our read mapping procedure to simulated RNA-seq reads from each population. Overall, the mapping efficiency was very high with ~98% of all simulated reads from both populations being mapped to the correct gene in the reference sequence (Table 4). The remaining reads either could not be mapped to the transcriptome (~0.02%) or were mapped to an incorrect gene (~2%). Incorrect mapping occurred mostly when the exonic content of a gene showed overlap with another gene or, in rare cases, when gene families consisted of closely related paralogs. Across all genes, European reads showed slightly, but not significantly, higher mapping efficiency (Table 4). A similar result was observed for the subsets of genes with significant over-expression in either Africa or Europe (Table 4). Given that the observed median difference in expression of significant genes between populations was 2.7-fold, the contribution of mapping bias to the observed expression differences is expected to be negligible.

**Table 4:** **Mapping efficiency of simulated RNA-seq reads to the reference *D*. *melanogaster *transcriptome**

		**Mean mapping efficiency in %**		
**Data set**	**Genes**	**Africa**	**Europe**	***P***
All genes	13,520	97.63 (9.73)	97.65 (9.45)	0.07
Africa over-expressed	110	98.33 (6.45)	98.40 (6.74)	0.48
Europe over-expressed	218	97.93 (8.00)	98.17 (6.49)	0.78

Standard deviations are given in parentheses. *P*-values are from Wilcoxon signed-ranks tests.

At the transcript level, we were able to identify 63 individual transcripts of multiple-transcript genes that differed in expression between the populations at an FDR of 5% (Additional file 4). The vast majority of these were cases where one transcript of a gene showed a significant bias towards one population and the other transcripts of that gene were either biased towards the same population or were not detected. Two transcripts of the gene *CHKov1* that are associated with a polymorphic transposable element insertion [34] showed significant over-expression in Europe (see below).

### Validation of RNA-seq results by qRT-PCR

**Figure 5:**
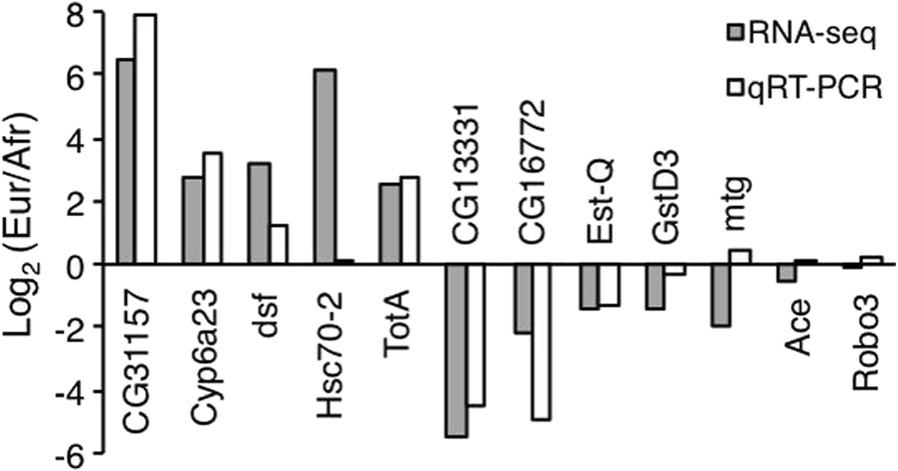
**Comparison of RNA-seq and qRT-PCR results.** The log_2_ ratio of European-to-African expression is shown for a subset of 12 genes that were measured by both RNA-seq (gray bars) and qRT-PCR (open bars).

For a subset of the genes analyzed by RNA-seq, we attempted to confirm the observed expression difference between populations using RNA extracted from new biological replicates and quantitative reverse-transcription PCR (qRT-PCR). The genes tested included five that were over-expressed in Europe (*CG31157*, *Cyp6a23, dsf*, *Hspc70-2*, and *TotA*), five that were over-expressed in Africa (*CG13331*, *CG16772*, *Est-Q*, *GstD3,* and *mtg*), and two that showed no difference in expression between the populations (*Ace* and *Robo3*). Overall, the expression ratios measured by the two methods were highly correlated (Pearson’s *R* = 0.79, *P* = 0.002). Qualitatively, all of the genes gave consistent results with the two methods, with the exception of *mtg*, which showed high over-expression in the African population by RNA-seq, but weak over-expression in the European population by qRT-PCR (Figure 5). The gene *Hsc70-2* showed European over-expression by both methods, however the magnitude of over-expression was much greater in the RNA-seq data (Figure 5). Otherwise, there was good agreement in the expression levels detected by RNA-seq and qRT-PCR (Additional file 5).

### Functional classification of differentially expressed genes

Of the genes expressed differentially between the European and African populations, six are known to be involved in the response to heat stress. These include the heat shock protein *Hsc70-2*, which showed 70-fold higher expression in European flies. A role for *Hsc70-2* in ecological adaptation is supported by parallel clines in genetic variation in both Australia and North America [35]. Another heat shock protein, *Hsp23*, showed two-fold over-expression in European flies. *Hsp23* is induced by both high and low temperatures [36,37], and its expression is associated with faster chill coma recovery [38], a phenotype known to differ between the African and European lines used in our analysis [39]. Two other genes that showed high over-expression in Europe, *TotA* (6-fold) and *TotC* (8-fold), are known to be involved in general stress response and show an induction in expression under both high and low temperatures [36,40].

A set of genes encoding chemosensory receptors was found to differ in expression between the populations. These included ionotropic glutamate receptors (*Ir93a* and *GluRIIA*), odorant-binding proteins (*Obp18a* and *Obp49a*), gustatory receptors (*Gr61a*), and olfactory receptors (*Or45b, Or63a, Or67d*, and *Or88a*). The four olfactory receptors were all expressed at higher levels (1.6–3.9-fold) in Europe than in Africa. *Or67d* binds to 11-*cis*-vaccenyl acetate, which is a volatile male-specific pheromone known to trigger aggregation and mating behavior in both sexes as well as male-male aggressive behavior [41-43]. *Or88a* is activated when flies are exposed to odors from virgin or mated females, although its exact ligand has not been identified [44].

The differentially expressed genes also included six glutathione S-transferase and seven cytochrome P450 genes, which are known to be important for detoxification. Notably, these included the cytochrome P450 gene *Cyp6g1*, whose over-expression is associated with resistance to DDT and related insecticides [45]. Previous studies of the same populations identified *Cyp6g1* as the gene with the greatest European over-expression when whole flies were examined [18,19]. In brain, *Cyp6g1* also shows strong over-expression (>4-fold) in European flies.

### A cluster of differentially expressed genes on chromosome arm 3R

**Figure 6:**
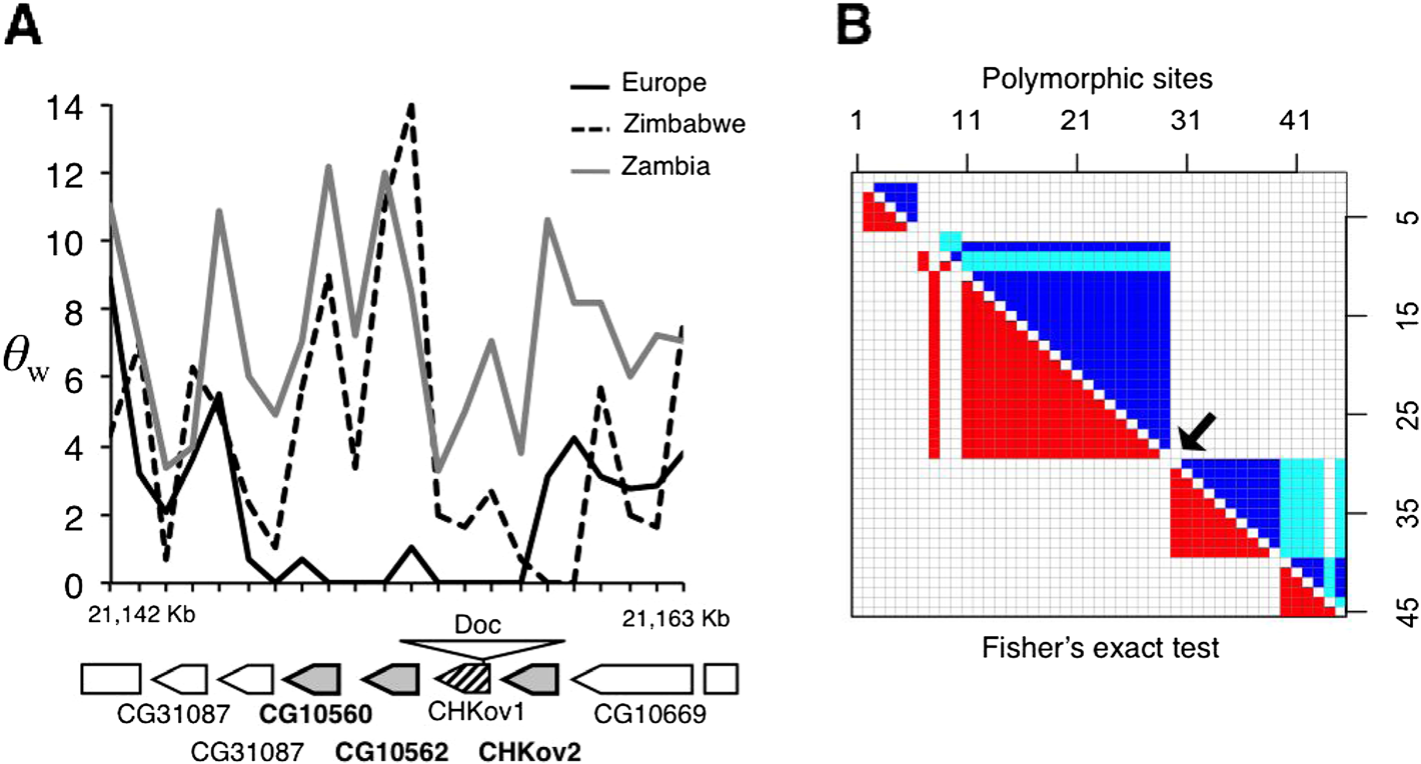
**A cluster of differentially expressed genes in a region of low nucleotide polymorphism and strong linkage disequilibrium within Europe.** (**A**) Nucleotide diversity (θ_W_) in two African populations (Zimbabwe, dashed line; Zambia, gray line) and a European population (the Netherlands, solid line) along a 22 kb region of chromosome 3R. The genes located in this region are depicted below the plot, with arrowheads indicating the direction of transcription. Gray boxes represent genes that are over-expressed in the African population. The hatched box represents *CHKov1*, which is disrupted by a *Doc* element insertion in all of the European lines. (**B**) Linkage disequilibrium between all pairs of polymorphisms (excluding singletons) within the same 22 kb region in the European population. Values of *r*^2^ are indicated in the upper matrix, with *r*^2^ ≤ 0.4 (white), 0.4 <*r*^2^ < 0.8 (light blue), and *r*^2^ ≥ 0.8 (dark blue). The lower matrix indicates the results of Fisher’s exact test, with *P* ≤ 0.05 (red) and *P* > 0.05 (white). The arrow indicates the location of the *CG10560*, *CG10562*, *CHKov1*, and *CHKov2* gene cluster.

Three genes that showed significant over-expression in the African population (*CG10560*, *CG10562*, and *CHKov2*) are located in a cluster on chromosome arm 3R. This cluster also contains the gene *CHKov1*, which is known to produce different transcript isoforms due to the presence/absence of a polymorphic *Doc* transposable element insertion [34]. All of these genes are predicted to encode choline kinases. We found that the *Doc* element insertion, which promotes transcription of *CHKov1* isoforms that exclude the choline kinase domain, was present in all 12 of our European lines, but only one of the 11 African lines (Additional file 6). This region of the genome shows a strong reduction in nucleotide polymorphism that is limited to the European population (Figure 6), which is consistent with a recent selective sweep. Furthermore, there are blocks of strong linkage disequilibrium (LD) on either side of the region of reduced polymorphism (Figure 6), as is expected in the case of a selective sweep [46]. To further test this, we computed the statistic *ω*, which quantifies LD on either side of a selected site relative to LD spanning the selected site [46,47]. High values of *ω* are expected following a selective sweep. The maximum value of *ω* in the *CHKov1* region was 33.95. A value this high occurred in only 7.8% of 10,000 neutral simulations that took into account the demographic history of the European population [48], indicating that the LD pattern is unlikely to be caused by demography alone.

## Discussion

RNA-seq analysis of dissected brains revealed over 300 genes that differ in expression between population samples from Africa and Europe. Importantly, the analysis of a single tissue uncovered many differentially expressed genes that were not found in previous studies that compared gene expression in whole flies from the same populations [18,19]. In total, the previous studies identified 708 genes that differed in expression between the African and European populations in either males [18] or females [19]. Of these, only 15 genes also differed significantly in brain expression between the two populations, with 11 showing the same direction of difference (i.e., African or European over-expression). The only gene common to all three data sets was the insecticide resistance gene, *Cyp6g1*, which always showed high over-expression (>3-fold) in European flies. Increased expression of *Cyp6g1* is associated with an *Accord* element insertion in its upstream region and DDT resistance [45]. This insertion is present in all of our European lines, but only in half of the African lines [19]. Although the *Accord* insertion upstream of *Cyp6g1* mainly affects its expression in midgut, Malpighian tubule, and fat body [49], our results suggest that the *Accord* insertion has a similar effect on *Cyp6g1* expression in the brain, where it is expressed at low levels. In contrast, the gene *CG9509*, which shows 2–3 times greater expression in whole flies from Europe than in those from Africa due to variation in a *cis*-regulatory sequence [50], did not differ significantly in its brain expression between the two populations. In this case, the between-population expression difference appears to be specific to the Malpighian tubules, where *CG9509* shows highly enriched expression [51].

Four choline kinase genes that differ in expression between the African and European populations are located in a 22-kb region of chromosome arm 3R that shows evidence for a recent selective sweep in non-African populations (Figure 6) [34,52]. Three of these genes (*CG10560, CG10562* and *CHKov2*) had significantly higher expression in Africa than in Europe. The fourth gene, *CHKov1*, did not differ in expression between populations in our gene-based analysis. However, it did differ in the transcript-based analysis. Two truncated *CHKov1* transcripts that do not contain the choline kinase domain and are associated with a *Doc* element insertion showed significant over-expression in the European population, while the full-length transcript showed strong (1.7-fold), but not significant, over-expression in Africa. This is consistent with our finding that the *Doc* element insertion is in high frequency in Europe (12 out of 12 lines), but low frequency in Africa (1 out of 11 lines). Taken together, these results suggest that selection has favored a reduction in the choline kinase activity of all four genes in the European population. Previous work has shown that the *Doc* element insertion in *CHKov1* is associated with increased resistance to an organophosphate pesticide [34] and to sigma virus [52]. However, it is not known if the *Doc* insertion itself was the target of selection, or if it has hitchhiked to high frequency due to linkage with another selected variant. It is also not known if the *Doc* insertion is responsible for the expression difference of all choline kinase genes in this region, or if other regulatory changes are involved. Although the *Doc* insertion in *CHKov1* and the *Accord* insertion upstream of *Cyp6g1* are both associated with insecticide resistance, a key difference is that the former is associated with reduced expression, while the latter is associated with increased expression. Thus, resistance to organophosphate and organochlorine insecticides appears to occur through different mechanisms with respect to gene regulation.

The proportion of genes that were found to be differentially expressed in brain between the African and European populations of *D*. *melanogaster* (~2%) is similar to what has been reported for comparisons of wild and domesticated populations of fish and mammals (~1%) [53,54]. In contrast, comparisons of brain transcriptomes between nursing and foraging bees [55] and male Atlantic salmon with different mating strategies [56] revealed much higher proportions of differentially expressed genes (15% and 35%, respectively). Although the number of differently expressed genes that is detected in an experiment is highly sensitive to the experimental design, replication scheme, pooling of samples, and method of statistical analysis, these findings suggest that divergence in brain gene expression may be greater when individuals are separated into two very distinct behavioral classes.

In contrast to studies using whole flies or heads [22,30,32,57,58], we detected relatively little sexual dimorphism in brain gene expression. The difference between brains and whole flies is expected, as the vast majority of genes showing sex-biased expression in whole flies are expressed in reproductive tissues [22]. In head, it appears that most sex-biased gene expression occurs outside of the brain. A previous microarray study found 754 sex-biased genes in the head, but only four in the central nervous system (brain plus ventral nerve cord) [58]. Similarly, an RNA-seq study identified 1,381 sex-biased genes in the head [32], while our RNA-seq analysis found only 91 sex-biased genes in the brain. These results suggest that nearly all of the sex-biased expression in the head occurs in non-nervous tissues such as the fat body, which is thought to play an important role in regulating sex-specific reproductive behavior and physiology [59].

Despite the relatively low level of sexual dimorphism in brain gene expression, we detected a significant over-representation of sex-biased genes (both male- and female-biased) on the X chromosome. Previous studies of whole flies observed a paucity of male-biased genes and an excess of female-biased genes on the X chromosome [22,57]. For male-biased genes, this pattern appears to be driven by gene expression in reproductive tissues [60] and an excess of X-linked male-biased genes in somatic tissue (head) has been reported [32,60]. It has been suggested that the over-representation of somatic male-biased genes on the X chromosome is related to the mechanism of dosage compensation [32]. Our data are consistent with this interpretation, as we find that X-linked, male-biased genes are significantly closer to MSL binding sites than female-biased or unbiased genes (Table 3). This suggests that their proximity to the MSL binding site leads to an “over-compensation” of expression in male somatic tissue. Our results contrast with those of a previous study of sex-biased gene expression in gonadectomized flies [61], which suggested that MSL binding might interfere with sex-specific regulation, leading to a reduction of male-biased expression. A possible explanation for this discrepancy is that the identification of sex-biased genes in gonadectomized flies might be confounded by variation in sex-bias among tissues. In comparison to male-biased genes, female-biased genes show a weaker enrichment on the X chromosome. Although there is some indication that X-linked female-biased genes tend to be located farther from MSL binding sites than male-biased or unbiased genes (Table 3), the sample size is too small to draw a firm conclusion. Thus, the excess of X-linked, female-biased genes may not be related to dosage compensation, but instead may reflect an overall feminization of the X chromosome [60], possibly caused by sexually antagonistic selection [21,62,63].

## Conclusions

Organisms may adapt to new or changing environmental conditions by altering levels of gene expression. Since expression profiles can vary greatly among tissues, it is likely that some adaptive regulatory changes are tissue- or organ-specific. For example, gene expression changes occurring specifically in the brain may underlie adaptive behavioral or physiological responses to the environment. However, there are few cases where gene expression and behavioral polymorphisms have been linked in an evolutionary framework [64] and more work is needed in order to understand the relationships among selection, gene expression, and behavior/physiology. To this end, we used RNA-seq to compare brain gene expression between two *D*. *melanogaster* populations from different habitats. In total, we identified 343 genes that differed in brain expression between the populations, the vast majority of which were not detected in analogous studies that used whole flies as the source of RNA.

Among the differentially expressed genes were those with functions in stress response, olfaction, and detoxification, including two genes previously implicated in insecticide resistance. Brain tissue is thought to be especially sensitive to heat, cold, and oxidative stress, and also can be affected by chemical stressors, such as insecticides. For example, some insects are known to metabolize insecticides specifically in nerve ganglia [65,66]. Thus, the alteration of gene expression in the brain may be particularly important for environmental adaptation. Further studies are needed to elucidate the contribution of specific gene expression changes to behavioral and physiological differences between populations and to determine the selective agents and regulatory mechanisms responsible for them.

## Methods

### Fly strains and brain dissection

The population samples consisted of 11 isofemale lines (*A84*, *A95*, *A131*, *A145*, *A157*, *A186*, *A191*, *A229*, *A377*, *A384*, and *A398*) collected from Lake Kariba, Zimbabwe and 12 isofemale lines (*E01*, *E02*, and *E11–E20*) collected from Leiden, the Netherlands [8,18,19]. An estimate of cosmopolitan admixture for our African population is not available. However, admixture estimates are available for two nearby populations, Siavonga, Zambia (9 km away) and Sengwa, Zimbabwe (33 km away) [67]. Both of these populations show admixture proportions below 3%, suggesting that admixture in our population should be negligible. All flies were maintained on standard cornmeal-molasses medium at 22° with a 14 h light:10 h dark cycle. Adult flies aged 2–4 days were anesthetized on ice and brains were dissected in 1xPBS (phosphate buffered saline) and stored in RNAlater (Qiagen) to prevent RNA degradation. Five or six brains from each of the African and European lines were dissected and pooled following the scheme shown in Figure 1. Two biological replicates were performed for each population and sex.

### RNA extraction and high-throughput sequencing

Total RNA extraction and DNase I digestion were performed using the MasterPure RNA Purification Kit (Epicentre). cDNA library construction and high-throughput sequencing were performed by GATC Biotech (Konstanz, Germany). Briefly, poly-A mRNA was purified and fragmented by sonication. First-strand, single-end cDNA was synthesized using random primers. Eight tagged libraries were generated, pooled and run on two lanes of a HiSeq 2000 sequencer (Illumina) to generate single reads of 50 bases. All sequences have been submitted to the GEO database under the series GSE40907.

### Read mapping

Illumina sequence reads were mapped to the reference *D*. *melanogaster* transcriptome (FlyBase release 5.43) [29] using Stampy (version 1.0.17) [68] with default parameters, except that expected divergence to the reference sequence was set to 1%. This divergence corresponds roughly to the upper limit of what is observed when comparing exonic regions of African sequences from the *Drosophila* Population Genomics Project (DPGP) [67] to the reference genome (0.5% – 0.7% divergence, depending on strain). For comparison, we also mapped all reads using Bowtie (version 0.12.7) [69] and allowing a maximum of three mismatches over the length of the sequence read (option –v 3). The two methods gave highly concordant results, with a nearly perfect correlation in the number of reads mapped per gene over all libraries (Pearson’s *R* > 0.99 in all cases). Overall, a higher proportion of reads were mapped to the transcriptome with Stampy (71%) than with Bowtie (68%). For this reason, we used the Stampy results for all subsequent analyses.

Two approaches were used to estimate expression levels. First, expression was quantified on a “per gene” basis. For this, if a sequence read mapped to one or more transcripts of the same gene, it was counted as one “hit” for that gene and all subsequent analyses were performed at the gene level. For calculations of RPKM, the length of the longest transcript of each gene was used. The use of the longest transcript systematically overestimates the true transcript length for genes with multiple transcripts, but is a reasonable compromise in situations where the relative abundance of the different transcript isoforms is unknown. In the second approach expression was quantified on a “per transcript” basis. For this, only reads that mapped uniquely to a single transcript were considered and all subsequent analyses were performed at the transcript level. Because there is high overlap among alternate transcript isoforms of the same gene, many reads could not be mapped to a specific transcript and were discarded. Thus, the “per transcript” approach results in a considerable loss of information. For this reason, all results are presented on a “per gene” basis unless otherwise noted. Reads that did not map to any transcript were mapped to other features of the *D*. *melanogaster* genome using annotation 5.43 and the procedure described above. For this, the single best match was used or, if there were multiple matches of equal quality, one was chosen arbitrarily. Reads that did not match any sequence in the genome were considered unmapped.

### Differential expression analysis

To test for differential expression between sexes or populations, we used the DESeq package (version 2.10) [70] implemented in R (version 2.14.1) [71]. This approach is based on the negative binomial distribution and accounts for the dispersion in read counts per gene across replicate samples. We analyzed data from all eight of our samples (Table 1) together using a two-factor design that included both population (Africa or Europe) and sex (male or female). Significant effects of population or sex were determined by comparing the fit of the two-factor model to that of a one-factor model that excluded the factor of interest using the function *fitNbinomGLMs*. The FDR was determined using Benjamini-Hochberg adjusted *P*-values [72]. A total of 2,390 genes with fewer than 16 mapped reads summed over all libraries, which was the minimum needed to detect significant differential expression under our experimental design, were removed prior to statistical analysis.

### Simulation analysis of mapping efficiency of African and European RNA-seq reads

To test for a potential population bias during the mapping process, we simulated RNA-seq samples using the whole genome sequences for three African (Zimbabwe) and eleven European (the Netherlands) strains made available by the DPGP as part of the DPGP2 data set (SRP005599) in the NCBI short read archive [67]. The African strains used were *A84*, *A131*, and *A186* (denoted by *ZK* in the DPGP data). The European strains used were *E01*, *E02*, and *E11E19*. For each transcript in the genome we randomly sampled 100 fragments of length 50 bp covering the exonic content for each available strain. Fragments that contained stretches of four or more consecutive uncalled or masked bases were removed from the sample, assuming the lack of data was the result of missing coverage during the sequencing and/or assembly process rather than indel polymorphism within coding regions. For some transcripts this quality control step resulted in the removal of all simulated reads for one or more strains. These transcripts were removed from the data set and a total of 13,520 genes located on the five major chromosomal arms remained for subsequent analysis. The simulated reads were then mapped to the reference transcriptome using Stampy [68] as described above and the proportion of fragments that could be mapped to the correct gene (referred to as mapping efficiency) was recorded for the African and European strains for each gene. For genes with multiple transcripts, mapping efficiencies were recorded for each transcript individually and then averaged to obtain per gene mapping efficiencies. The above analysis was repeated for the subset of genes detected as differentially expressed between populations in our RNA-seq experiment, but the number of sampled fragments was increased to 10,000 in order to improve the sensitivity of our method. Of the 343 genes in this data set, six were removed because they were not located on one of the five major chromosomal arms and another nine genes, all being part of the European over-expressed subset, were removed by the quality control step that excluded reads containing too many consecutive uncalled bases. All of these latter nine genes were located either close to the centromere or in regions containing DNA duplications, which can explain the lack of properly assembled genomic sequence. This lack of data did not show a population bias, as it was found in both European and African strains for all nine genes.

### qRT-PCR

On the basis of the RNA-seq data, 12 genes were chosen for qRT-PCR analysis. Brain dissection, sample pooling, and total RNA extraction were performed as described above for RNA-seq, with the exception that four biological replicates were performed for each population and sex. cDNA was synthesized using random primers and SuperScript II reverse transcriptase (Invitrogen) according to the manufacturer’s instructions. TaqMan Gene Expression Assays (Applied Biosystems) were used for the following genes (TaqMan IDs are given in parentheses): *Ace* (Dm02134758_g1), *robo3* (Dm01800570_g1), *Hsc70-2* (Dm02330923_gH), *dsf* (Dm01842631_g1), *TotA* (Dm02363547_s1), *Cyp6a23* (Dm01824231_g1), *CG31157* (Dm02148979_g), *GstD3* (Dm02153755_s1), *Est-Q* (Dm01792292_g1), *mtg* (Dm02137882_g1), *CG13331* (Dm01797351_g1), and *CG16772* (Dm02365807_s1). The ribosomal protein gene *RpL32* (Dm02151827_g1) was used as a reference gene for expression normalization. qRT-PCR was performed using a Real-Time thermal cycler CFX96 (Bio-Rad). The ΔΔCt method was used to compute the normalized expression of the genes of interest [73].

### PCR assay for the *Doc* element insertion in *CHKov1*

Specific forward (5’-GAACTCCGTGGGATCGACTA-3’) and reverse (5’-GCGGAGCTTTTGAGAGAAGA-3’) primers were designed to detect the presence or absence of the *Doc{}CHKov1*^*Doc1420*^ transposable element insertion in *CHKov1.* The 23 isofemale lines from the African and European populations (described above) were tested for the presence of the *Doc* element. DNA was extracted from a single fly of each line using the MasterPure DNA Purification Kit (Epicentre). PCR was conducted under standard conditions and the presence or absence of the *Doc* element was determined by the size of the amplified fragment (5.5 kb with the insertion, 1 kb without) as determined by 1% agarose gel electrophoresis.

### Population genetic analysis

To analyze DNA sequence polymorphism in the *CHKov1* region, we used whole genome sequences generated by the DPGP [67]. The European population sample consisted of 11 of the Netherlands lines used in our RNA-seq analysis. One African population sample consisted of three of the Zimbabwe lines used in our RNA-seq analysis, while a second African population sample consisted of four lines from Siavonga, Zambia. Nucleotide diversity was calculated using Watterson’s estimator, θ_W_[74]. Linkage disequilibrium (LD) between any two polymorphic sites was calculated using Lewontin’s *r*^2^= *D*^2^/*p*_1_*q*_1_*p*_2_*q*_2_ where *D* is the frequency of the haplotypes and *p* and *q* represent the allele frequencies [75]. We calculated *r*^2^ between all pairs of polymorphic sites, excluding singletons. Significance of the *r*^2^ values was assessed with Fisher’s exact test. The *ω* statistic was calculated using the sliding window approach implemented in the software OmegaPlus [47], with a minimum of four polymorphisms per window. To determine if the observed *ω* value could be expected under a neutral non-equilibrium demographic model, we conducted a parametric bootstrap analysis [76]. For this, we generated 10,000 simulated data sets using a coalescent demographic model that takes into account our current knowledge of the demographic history of the African and European populations of *D*. *melanogaster*[48]. This model assumes that the African and European populations diverged 128,430 generations ago from an ancestral population with an effective size (*N*_e_) of 1,705,328. At the time of divergence, the European *N*_e_ was reduced to 32,128 before directly entering a phase of exponential growth until reaching a current *N*_e_ of 878,506. The simulated data sets were identical to the observed data set in terms of mutation rate, recombination rate, number of sites, and number of sampled individuals. For every simulated data set we performed a sliding-window analysis of the *ω* statistic and recorded the maximal value. The *P*-value was defined as the proportion of simulations with *ω* greater than or equal to the observed value.

## Competing interests

The authors declare that they have no competing interests.

## Authors' contributions

AC and JP conceived of the study and its design. AC performed the brain dissections, RNA extractions and qRT-PCR. AC, SH and JP analyzed the RNA-seq data. AC and SH performed the population genetic analyses. AC and JP wrote the manuscript with input from SH. All authors read and approved the final manuscript.

## Supplementary Material

Additional file 1 **Expression levels of all genes in all replicates**. Table of read counts and relative expression levels of all genes in all replicates, including *P*-values for comparisons between populations and sexes. Click here for file

Additional file 2 **Sex-biased gene expression in the brain,** Table of genes showing significant sex-biased gene expression in the brain and their expression bias in whole flies. Click here for file

Additional file 3 **Transcripts of multiple-transcript genes that differ in expression between the sexes.** Table of individual transcripts that show significant sex-biased expression. Click here for file

Additional file 4 **Transcripts of multiple-transcript genes that differ in expression between populations.** Table of individual transcripts that show a significant expression difference between the African and European populations. Click here for file

Additional file 5 **Results of qRT-PCR.** Figure showing the relative expression of genes in the African and European populations as determined by qRT-PCR. Click here for file

Additional file 6 **PCR assay for the*Doc*element insertion in*CHKov1.*** Figure showing the results of the PCR assay to detect the presence of the *Doc* element insertion in *CHKov1* in all African and European lines. Click here for file
