# Supplementary material for: Population and sex differences in Drosophila melanogaster brain gene expression
Source: BMC Genomics. 2012 Nov 21;13:654. doi: 10.1186/1471-2164-13-654 (PMC3527002; doi:10.1186/1471-2164-13-654)
Supplement: Additional file 2 — Sex-biased gene expression in the brain, Table of genes showing significant sex-biased gene expression in the brain and their expression bias in whole flies. [file 1471-2164-13-654-S2.pdf]

Additional file 2: Sex-biased gene expression in the brain

		Sex bias		
Gene	Symbol	Brain	Whole fly[a]	Whole fly[b]
CG7024	*CG7024*	Male	Male	Male
CG9806	*CG9806*	Male	Male	Male
CG7592	*Obp99b*	Male	Male	Male
CG4979	*sxe2*	Male	Male	Male
CG6999	*CG6999*	Male	Male	Male
CG3360	*Cyp313a1*	Male	Male	Male
CG6730	*Cyp4d21*	Male	Male	Male
CG11315	*Npc2h*	Male	Male	Male
CG17052	*obst-A*	Male	Male	Male
CG13057	*retinin*	Male	Male	Male
CG3699	*CG3699*	Male	Male	Male
CG3466	*Cyp4d2*	Male	Male	Male
CG32706	*CG32706*	Male	Male	NA
CG2681	*CG2681*	Male	Male	Unbiased
CG14787	*CG14787*	Male	Male	Unbiased
CG13762	*CG13762*	Male	Male	Unbiased
CG6788	*CG6788*	Male	Male	Unbiased
CG32673	*Rab9E*	Male	Male	Unbiased
CG42398	*SteXh:CG42398*	Male	Male	Unbiased
CG11648	*Abd-B*	Male	Male	Unbiased
CG14195	*CG14195*	Male	Male	Unbiased
CG18467	*CG18467*	Male	Male	Unbiased
CG3540	*Cyp4d14*	Male	Male	Unbiased
CG40305	*FucTC*	Male	Male	Unbiased
CG3630	*CG3630*	Male	Male	Unbiased
CG2709	*CG2709*	Male	Unbiased	Unbiased
CG2706	*fs(1)Yb*	Male	Unbiased	Unbiased
CG4872	*CG4872*	Male	Unbiased	Unbiased
CG2904	*ec*	Male	Unbiased	Unbiased
CG3706	*CG3706*	Male	Unbiased	Unbiased
CG9307	*Cht5*	Male	Unbiased	Unbiased
CG4542	*CG4542*	Male	Unbiased	Female
CG32763	*l(1)G0045*	Male	Female	Unbiased
CG4293	*CG4293*	Male	Female	Female
CG9904	*CG9904*	Male	Female	Female
CG6461	*CG6461*	Male	Female	Female
CG15914	*CG15914*	Male	Female	Female
CG10803	*CG10803*	Male	Female	Female
CG9203	*CG9203*	Male	Female	Female
CG8939	*CG8939*	Male	Female	Female
CG9938	*Ndc80*	Male	Female	Female
CG2079	*Dok*	Male	Female	Female
CG1994	*l(1)G0020*	Male	Female	Female
CG14814	*CG14814*	Male	Female	Female
CG11164	*CG11164*	Male	Female	Female
CG4206	*Mcm3*	Male	Female	Female
CG4586	*CG4586*	Male	Female	Female
CG11420	*png*	Male	Female	Female
CG3692	*CalpC*	Male	Female	Female
CG2979	*Yp2*	Female	Female	Female
CG11129	*Yp3*	Female	Female	Female
CG4790	*fs(1)M3*	Female	Female	Female

CG2985	*Yp1*	Female	Female	Female
CG17820	*fit*	Female	Female	Female
CG9772	*CG9772*	Female	Female	Female
CG2054	*Cht2*	Female	Female	Female
CG2003	*CG2003*	Female	Female	Female
CG6806	*Lsp2*	Female	Female	Unbiased
CG14645	*CG14645*	Female	Female	Unbiased
CG4020	*CG4020*	Female	Unbiased	Female
CG31077	*CG31077*	Female	Unbiased	Unbiased
CG9743	*CG9743*	Female	Unbiased	Unbiased
CG3027	*pyd3*	Female	Unbiased	Unbiased
CG2962	*CG2962*	Female	Unbiased	Unbiased
CG14167	*Ilp3*	Female	Unbiased	Unbiased
CG9280	*Glt*	Female	Unbiased	Unbiased
CG15308	*CG15308*	Female	Unbiased	Unbiased
CG6667	*dl*	Female	Unbiased	Unbiased
CG4950	*CG4950*	Female	Unbiased	Unbiased
CG3757	*y*	Female	Unbiased	Unbiased
CG8942	*nimC1*	Female	Unbiased	Unbiased
CG4786	*Rcd2*	Female	Unbiased	Unbiased
CG3823	*CG3823*	Female	Unbiased	Unbiased
CG7002	*Hml*	Female	Male	Unbiased
CG4797	*CG4797*	Female	Male	Unbiased
CG42639	*proPO-A1*	Female	Male	Unbiased
CG33103	*Ppn*	Female	Male	Unbiased
CG6698	*NtR*	Female	Male	Unbiased
CG4099	*Sr-CI*	Female	Male	Unbiased
CG33273	*Ilp5*	Female	Male	Unbiased
CG34324	*CG34324*	Female	Male	Unbiased
CG15279	*CG15279*	Female	Male	Male
CG3906	*CG3906*	Female	Male	Male
CG14125	*CG14125*	Female	Male	Male
CG14277	*CG14277*	Female	Male	Male
CG13360	*CG13360*	Female	Male	Male
CG14456	*CG14456*	Female	Male	Male
CG15459	*CG15459*	Female	Male	Male
CG7106	*lectin-28C*	Female	Male	Male
CG34220	*CG34220*	Female	Male	NA
CG43673	*CG43673*	Female	Male	NA

[a]Data from modENCODE [31].

[b]Data from Sebida meta-analysis (v. 3) [30].
