# Supplementary material for: Population and sex differences in Drosophila melanogaster brain gene expression
Source: BMC Genomics. 2012 Nov 21;13:654. doi: 10.1186/1471-2164-13-654 (PMC3527002; doi:10.1186/1471-2164-13-654)
Supplement: Additional file 3 — Transcripts of multiple-transcript genes that differ in expression between the sexes. Table of individual transcripts that show significant sex-biased expression. [file 1471-2164-13-654-S3.pdf]

Additional file 3: Transcripts of multiple-transcript genes that differ in expression between the sexes

Transcript	Gene	Bias	Male/Female	Adj. *P*
FBtr0075364	*tra*	Female	0	1.97E-08
FBtr0081759	*dsx*	Male	4.09628443	8.14E-08
FBtr0083384	*Abd-B*	Male	7.73982604	8.14E-08
FBtr0305966	*Cht2*	Female	0.29785654	9.99E-05
FBtr0100476	*CG4293*	Male	9.84391044	0.00036357
FBtr0070982	*RpL17*	Female	0.12628794	0.00153393
FBtr0071136	*RpS6*	Female	0.24282851	0.00183333
FBtr0307287	*lawc*	Female	0.3621625	0.00537838
FBtr0071035	*inx7*	Female	0.28913877	0.0112404
FBtr0073576	*regucalcin*	Female	0.41537879	0.04654299
