# Supplementary material for: Population and sex differences in Drosophila melanogaster brain gene expression
Source: BMC Genomics. 2012 Nov 21;13:654. doi: 10.1186/1471-2164-13-654 (PMC3527002; doi:10.1186/1471-2164-13-654)
Supplement: Additional file 4 — Transcripts of multiple-transcript genes that differ in expression between populations. Table of individual transcripts that show a significant expression difference between the African and European populations. [file 1471-2164-13-654-S4.pdf]

**Additional file 4: Transcripts of multiple-transcript genes that differ in expression between populations**

| Transcript | Gene | Bias | Eur/Afr | Adj. *P* |
|---|---|---|---|---|
| FBtr0113212 | *CG12947* | Europe | 11.5055802 | 0 |
| FBtr0089939 | *Sod3* | Europe | 39.5166675 | 0 |
| FBtr0085427 | *CG2010* | Africa | 0.05405252 | 2.36E-10 |
| FBtr0113213 | *CG12947* | Europe | 8.77504172 | 2.36E-10 |
| FBtr0100496 | *CG13430* | Europe | 99.99999999 | 6.84E-09 |
| FBtr0073556 | *Karl* | Africa | 0.04130866 | 3.42E-08 |
| FBtr0303709 | *CG42797* | Europe | 12.46487 | 1.33E-07 |
| FBtr0113353 | *CHKov1* | Europe | 4.30716314 | 2.48E-07 |
| FBtr0073557 | *Karl* | Africa | 0.16611369 | 9.25E-07 |
| FBtr0301854 | *CG34423* | Europe | 6.27349023 | 9.51E-07 |
| FBtr0299720 | *Ir93a* | Europe | 13.2747227 | 1.11E-06 |
| FBtr0305582 | *Ag5r* | Europe | 5.14790009 | 1.30E-06 |
| FBtr0086900 | *RhoGAP54D* | Europe | 3.88335571 | 2.49E-06 |
| FBtr0078118 | *CG11455* | Europe | 3.16993035 | 3.10E-06 |
| FBtr0074365 | *Sep4* | Europe | 6.96126718 | 3.76E-06 |
| FBtr0077540 | *CG31955* | Europe | 4.84346749 | 4.07E-06 |
| FBtr0074186 | *CG8974* | Africa | 0.23442985 | 4.63E-06 |
| FBtr0076527 | *Tequila* | Europe | 2.92458226 | 0.00010193 |
| FBtr0299561 | *Cpr62Ba* | Africa | 0.14923376 | 0.00012116 |
| FBtr0099994 | *CG33958* | Africa | 0.25373311 | 0.0001363 |
| FBtr0308624 | *scrib* | Europe | 5.84416268 | 0.00029838 |
| FBtr0306906 | *Klp54D* | Europe | 3.50297321 | 0.00031137 |
| FBtr0306234 | *lok* | Europe | 99.99999999 | 0.00037285 |
| FBtr0307170 | *CG14985* | Europe | 40.9845856 | 0.00118455 |
| FBtr0084139 | *CG6656* | Europe | 2.88882775 | 0.0016121 |
| FBtr0077538 | *CG2818* | Europe | 3.86592944 | 0.0026213 |
| FBtr0100658 | *Sod3* | Europe | 2.37640694 | 0.00279063 |
| FBtr0308224 | *mRpL12* | Africa | 0.34018592 | 0.00450618 |
| FBtr0299586 | *fz2* | Africa | 0.40735149 | 0.00623311 |
| FBtr0302847 | *Gfat1* | Africa | 0.30290828 | 0.00623311 |
| FBtr0113351 | *CHKov1* | Europe | 2.51571059 | 0.006266 |
| FBtr0290031 | *Vha44* | Africa | 0.07125104 | 0.00639859 |
| FBtr0084101 | *CG3301* | Europe | 26.1226508 | 0.00893565 |
| FBtr0307389 | *fau* | Europe | 3.96744765 | 0.00900808 |
| FBtr0301887 | *CG11455* | Europe | 2.39466239 | 0.01082625 |

| | | | | |
|---|---|---|---|---|
| FBtr0077243 | *Mgstl* | Europe | 2.10821709 | 0.01093632 |
| FBtr0112717 | *Snoo* | Europe | 3.47495816 | 0.01270455 |
| FBtr0304846 | *Octbeta2R* | Europe | 2.67054076 | 0.01287655 |
| FBtr0084858 | *CG10550* | Europe | 3.7322172 | 0.01406873 |
| FBtr0100343 | *sv* | Africa | 0.0642603 | 0.01668524 |
| FBtr0078084 | *cbt* | Europe | 3.51599084 | 0.01670706 |
| FBtr0112927 | *Trf2* | Europe | 3.45847601 | 0.01670706 |
| FBtr0302130 | *Pde8* | Africa | 0.37089452 | 0.01695904 |
| FBtr0309042 | *sls* | Europe | 2.48208772 | 0.0171988 |
| FBtr0076114 | *Bmcp* | Europe | 3.29602191 | 0.02127831 |
| FBtr0301433 | *CG31279* | Africa | 0 | 0.02133633 |
| FBtr0082557 | *KLHL18* | Africa | 0.32758103 | 0.02228499 |
| FBtr0308340 | *CG33521* | Europe | 3.09209872 | 0.02529823 |
| FBtr0074659 | *bnb* | Africa | 0.1721355 | 0.02686506 |
| FBtr0113101 | *CG10320* | Europe | 2.26550063 | 0.03103432 |
| FBtr0301823 | *Mnt* | Europe | 2.57736925 | 0.03247037 |
| FBtr0088792 | *pnut* | Europe | 2.92656826 | 0.03324336 |
| FBtr0305994 | *l(2)06225* | Europe | 3.16660201 | 0.03437594 |
| FBtr0087106 | *RpLP2* | Europe | 5.5665086 | 0.03991185 |
| FBtr0085847 | *CG1971* | Africa | 0.08854299 | 0.03991185 |
| FBtr0076511 | *mRpL12* | Europe | 2.83526372 | 0.03991185 |
| FBtr0077342 | *fog* | Africa | 0.3211506 | 0.03991185 |
| FBtr0073947 | *CG14411* | Europe | 12.1139135 | 0.04119429 |
| FBtr0073690 | *CG3775* | Europe | 11.5409369 | 0.04119429 |
| FBtr0070362 | *trr* | Europe | 2.72418524 | 0.04236765 |
| FBtr0299929 | *sqa* | Africa | 0.23500644 | 0.04525254 |
| FBtr0079777 | *CG31708* | Africa | 0.21417141 | 0.04664426 |
| FBtr0089712 | *CG1677* | Europe | 2.08470231 | 0.04664426 |
