# Supplementary material for: Population and sex differences in Drosophila melanogaster brain gene expression
Source: BMC Genomics. 2012 Nov 21;13:654. doi: 10.1186/1471-2164-13-654 (PMC3527002; doi:10.1186/1471-2164-13-654)
Supplement: Additional file 5 — Results of qRT-PCR. Figure showing the relative expression of genes in the African and European populations as determined by qRT-PCR. [file 1471-2164-13-654-S5.pdf]

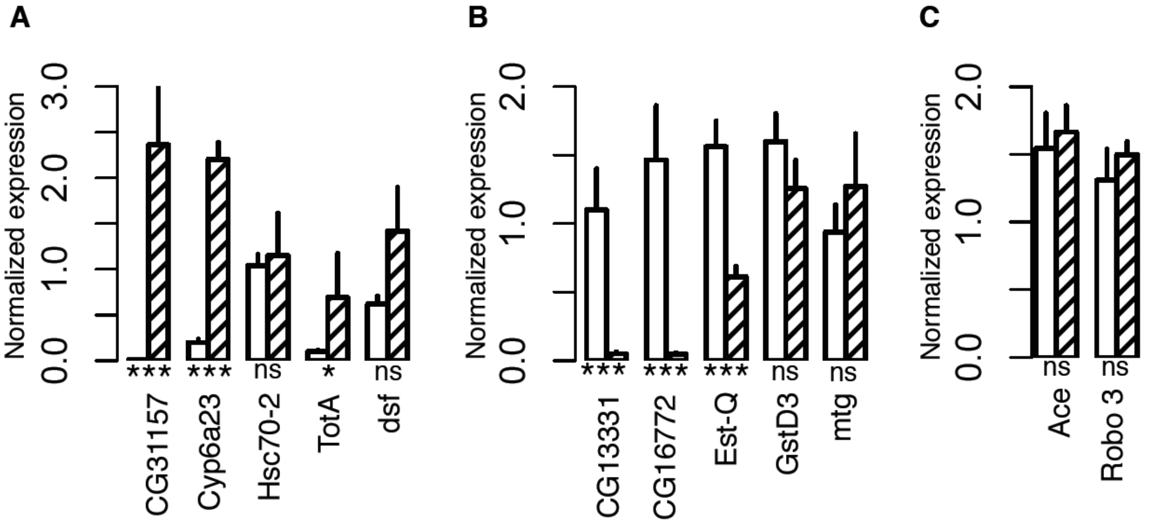

Additional file 5 - Results of qRT-PCR

The relative expression in Africa (open bars) and Europe (hatched bars) for genes tested by qRT-PCR. (A) Genes showing European over-expression in the RNA-seq analysis. (B) Genes showing African over-expression in the RNA-seq analysis. (C) Control genes showing equal expression in Europe and Africa in the RNA-seq analysis. Differences between population were tested with a Wilcoxon test. **P* < 0.05, ***P* < 0.001, ****P* < 0.0001, ns, not significant.
