# Supplementary material for: Population and sex differences in Drosophila melanogaster brain gene expression
Source: BMC Genomics. 2012 Nov 21;13:654. doi: 10.1186/1471-2164-13-654 (PMC3527002; doi:10.1186/1471-2164-13-654)
Supplement: Additional file 6 — PCR assay for theDocelement insertion inCHKov1. Figure showing the results of the PCR assay to detect the presence of the Doc element insertion in CHKov1 in all African and European lines. [file 1471-2164-13-654-S6.pdf]

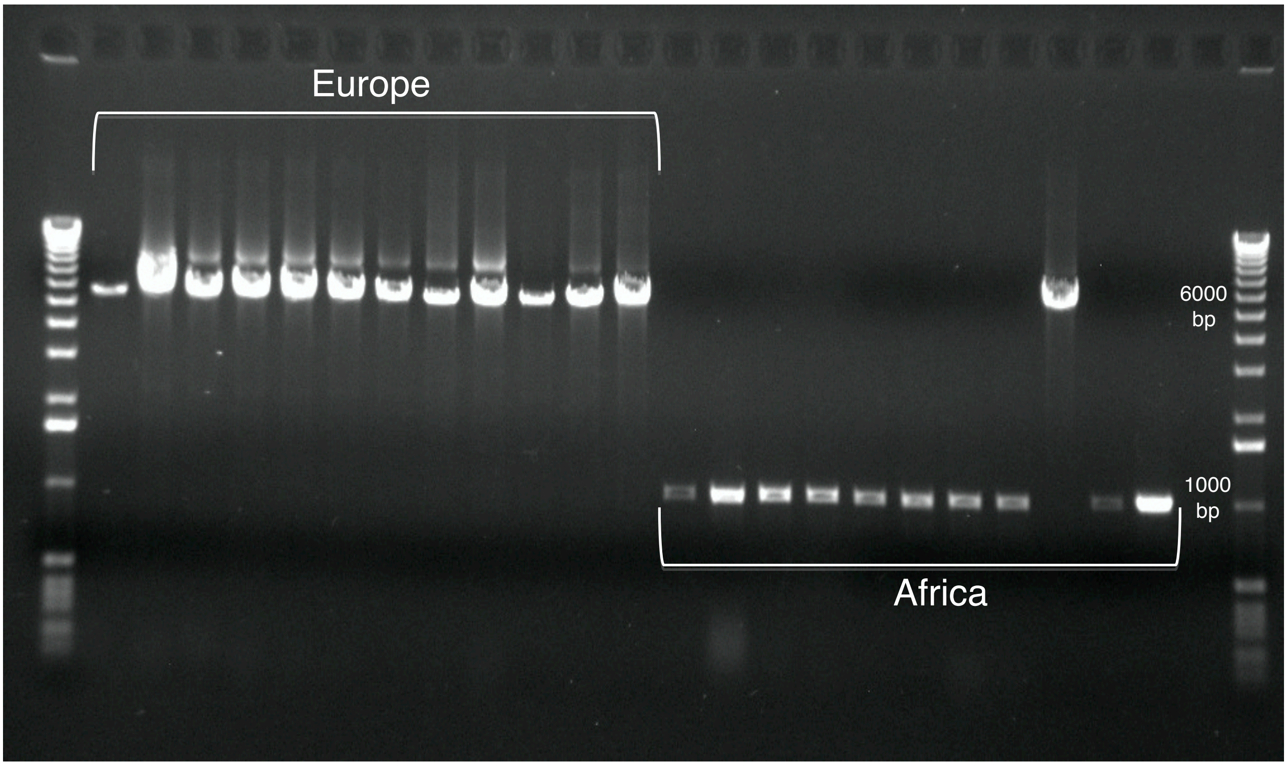

Additional file 6 - PCR assay for the *Doc* element insertion in *CHKov1*

The 12 European and 11 African lines used in the expression analysis were tested for the *Doc* element in *CHKov1* by PCR using primers that span the insertion site. The presence of the *Doc* element results in a PCR product of 5.5 kb, while its absence results in a product of 1 kb.
